# Fine mapping a QTL for BYDV-PAV resistance in maize

**DOI:** 10.1007/s00122-024-04668-z

**Published:** 2024-06-19

**Authors:** Maria Schmidt, Ricardo Guerreiro, Nadia Baig, Antje Habekuß, Torsten Will, Britta Ruckwied, Benjamin Stich

**Affiliations:** 1 Institute for Quantitative Genetics and Genomics of Plants, Heinrich Heine University, Düsseldorf, Germany; 2 Federal Research Center for Cultivated Plants, Institute for Resistance Research and Stress Tolerance, Julius-Kühn Institute, Quedlinburg, Germany; 3 Cluster of Excellence On Plant Sciences, From Complex Traits Towards Synthetic Modules, Heinrich Heine University, Düsseldorf, Germany; 4 Federal Research Center for Cultivated Plants, Institute for Breeding Research On Agricultural Crops, Julius-Kühn Institute, Sanitz, Germany

## Abstract

Barley yellow dwarf (BYD) is one of the economically most important virus diseases of cereals worldwide, causing yield losses up to 80%. The means to control BYD are limited, and the use of genetically resistant cultivars is the most economical and environmentally friendly approach. The objectives of this study were i) to identify the causative gene for BYD virus (BYDV)-PAV resistance in maize, ii) to identify single nucleotide polymorphisms and/or structural variations in the gene sequences, which may cause differing susceptibilities to BYDV-PAV of maize inbreds, and iii) to characterize the effect of BYDV-PAV infection on gene expression of susceptible, tolerant, and resistant maize inbreds. Using two biparental mapping populations, we could reduce a previously published quantitative trait locus for BYDV-PAV resistance in maize to ~ 0.3 Mbp, comprising nine genes. Association mapping and gene expression analysis further reduced the number of candidate genes for BYDV-PAV resistance in maize to two: Zm00001eb428010 and Zm00001eb428020. The predicted functions of these genes suggest that they confer BYDV-PAV resistance either via interfering with virus replication or by inducing reactive oxygen species signaling. The gene sequence of Zm00001eb428010 is affected by a 54 bp deletion in the 5`-UTR and a protein altering variant in BYDV-PAV-resistant maize inbreds but not in BYDV-PAV-susceptible and -tolerant inbreds. This finding suggests that altered abundance and/or properties of the proteins encoded by Zm00001eb428010 may lead to BYDV-PAV resistance.

**Supplementary Information:**

The online version contains supplementary material available at 10.1007/s00122-024-04668-z.

## Introduction

World food consumption heavily relies on cereals. The three most important food crops in the world are rice, wheat, and maize (corn), accounting for about 42% of the world’s human food calorie intake and 37% of protein intake (Erenstein et al. 2022). With an expected increase in the world population (United Nations 2022), an increasing demand for cereals is expected (OECD/FAO 2023). However, climate change effects, herbivore pests and several fungal, bacterial, and viral diseases threaten global cereal production (Rivero et al. 2022; Savary et al. 2019).

Barley yellow dwarf (BYD) is one of the economically most important diseases in small-grain cereals (Choudhury et al. 2017; van den Eynde et al. 2020). It can infect all members of the grass family (*Poaceae*), causing yield losses in cereals up to 80% but also negatively affecting grain quality (Choudhury et al. 2019; Nancarrow et al. 2021; Peiris et al. 2019). BYD symptoms vary between species, variety, and environmental conditions. In maize, red bands at the leaf edges and interveinal flecking of leaves are most common (Beuve et al. 1999; Brown et al. 1984; Grüntzig et al. 1997; Pearson and Robb 1984; Osler et al. 1985; Stoner 1977). A reduction of plant height, ear height, and biomass, as well as an earlier flowering, was observed in BYD-infected maize compared to non-inoculated maize plants (Beuve et al. 1999; Loi et al. 2004; Panayotou 1977). Grain yield losses up to 20% and dry matter yield losses up to 50% were reported (Beuve et al. 1999; Pearson and Robb 1984). Additionally, BYD in maize also changes dry matter content, metabolisable energy, digestible crude protein, and water-soluble carbohydrates (Pearson and Robb 1984).

At least ten different phloem-limited single-stranded, positive sense RNA viruses called Barley yellow dwarf viruses (BYDV) and cereal yellow dwarf viruses (CYDV) cause BYD (Walls et al. 2019; Miller and Lozier 2022). BYDVs and CYDVs are transmitted by more than 25 aphid species worldwide (Halbert and Voegtlin 1995). Worldwide, BYDV-PAV is the most prevalent virus species causing BYD, mainly transmitted by *Rophalosiphum padi* (Aradottir and Crespo-Herrera 2021). Several studies suggest that climate change will promote the spread of *R. padi* and, therefore, of BYDV-PAV (for review, see Irwin and Thresh 1990; Moreno-Delafuente et al. 2020).

Viruses possess no metabolism of their own, hampering their direct control. Insecticides to limit vector spread are not desirable because of cost and potential environmental damage (Chagnon et al. 2015; Serrão et al. 2022). Due to their harmful effects on beneficial insects, neonicotinoids, a very efficient and previously widely used insecticide class (Simon-Delso et al. 2015), are banned in the European Union (Regulation (EU) No. 485/2013). Moreover, resistance against pyrethroid insecticides is evident in some *R. padi* populations (Walsh et al. 2020). Thus, genetically resistant cereal cultivars are the most promising approach to limit the spread of BYD.

However, the unavailability of reliable high-throughput phenotyping methods hinders breeding for BYD resistance. On one hand, BYD symptoms are influenced by the environment and might be confounded with symptoms of other diseases, nutrient deficiency, waterlogging or mechanical injury (Grüntzig et al. 1997). On the other, BYD-tolerant genotypes show no symptoms, but the virus can replicate and move systemically in these plants. Thus, BYD-tolerant plants act as a virus reservoir and are a source for infection of other cereals. In contrast, resistant plants inhibit virus replication and/or systemic movement.

A more reliable alternative to visual symptom scoring is quantifying the virus titer in plants by quantitative real-time PCR, double antibody sandwich enzyme-linked immunosorbent assay (DAS-ELISA), or tissue blot immunoassay (Canning et al. 1996; Chéour et al. 1993; Choudhury et al. 2018). However, these methods are, from a breeder’s perspective, laborious and time-consuming. Thus, molecular markers that are at least closely linked to the resistance gene will help to accelerate the breeding process for BYD-resistant varieties.

Maize plays an important role in the BYD transmission cycle, serving as a “green bridge” between harvesting small-grain cereals in early summer and sowing of winter cereals in autumn (Brown et al. 1984; Haack et al. 1999; Rashidi et al. 2021). This effect is intensified by increasing viruliferous aphid populations under global warming conditions (Moreno-Delafuente et al. 2020).

To date, no broad-spectrum resistance against BYD is known in wheat and barley (for reviews, see Aradottir and Crespo-Herrera 2021; Choudhury et al. 2017; Jarŏsová et al. 2016; Walls et al. 2019). Thus, cultivating BYD-resistant maize is expected to reduce BYD pressure on not only maize but also small-grain cereals, such as wheat and barley, by breaking the BYD infection cycle.

BYDV-PAV resistance in maize shows high genotypic variance and heritability (Horn et al. 2013, 2014, and 2015), making it a promising target for breeding efforts. BYD-tolerant and -resistant maize genotypes were identified previously (Brown et al. 1984; Grüntzig and Fuchs 2000; Horn et al. 2014; Loi et al. 1986; Osler et al. 1985; Stoner 1977). Recently, a quantitative trait locus (QTL) for BYDV-PAV resistance was discovered in maize on the distal end of chromosome 10 (Horn et al. 2014 and 2015). In a genome-wide association study, Horn et al. (2014) identified three single nucleotide polymorphisms (SNPs) in gene GRMZM2G018027, which were associated with BYDV-PAV resistance. These SNPs explained between 16 and 21% of the phenotypic variance of the trait virus titer (EX) as well as between 11 and 18% of the phenotypic variance of the trait infection rate (IR). Similarly, in another study employing five connected linkage mapping populations, Horn et al. (2015) identified a QTL on the distal end of chromosome 10 which overlapped with the above described gene GRMZM2G018027 and explained 45% of the phenotypic variance for the traits EX and IR. However, the causative gene underlying this QTL was unidentified.

The objectives of this study were i) to identify the causative gene for BYDV-PAV resistance in maize, ii) to identify SNPs and/or structural variations in the gene sequences of maize inbreds, which may cause different susceptibilitties to BYDV-PAV, and iii) to characterize the effect of BYDV-PAV infection on gene expression of susceptible, tolerant, and resistant maize inbreds. The findings may be used to develop markers for marker-assisted breeding of BYDV-PAV-resistant maize and as a starting point to investigate the resistance mechanism. However, the cloning of this BYDV-PAV resistance QTL will be also informative for the breeding of BYD-resistant barley and wheat genotypes as targets for mutagenesis experiments.

## Methods

### Plant cultivation and aphid rearing

Maize plants (*Zea mays* L.) were grown in a greenhouse (16 h light, 20 °C/8 h darkness, 16 °C) for phenotyping of segregating material or in a climate chamber (16 h light, 24 °C/8 h darkness, 22 °C) for all other experiments.

BYDV-PAV carrying and virus-free aphids of species *Rhopalosiphum padi* were obtained from the collection of the Julius Kühn-Institute, Institute for Resistance Research and Stress Tolerance. The aphids were reared on BYDV-susceptible barley cv. “Rubina” at room temperature under artificial light conditions. Viruferous and virus-free aphids were checked regularly for BYDV-PAV, using the DAS-ELISA method with in-house polyclonal antisera for BYDV-PAV from the Julius Kühn-Institute as described by Horn et al. (2013).

#### Fine mapping of the BYDV-PAV resistance gene

### Plant material

Our study was based on heterogenous inbred family (HIF) populations developed from recombinant inbred lines (RILs) derived from crosses of BYDV-PAV tolerant inbred line P092 with BYDV-PAV resistant inbred lines FAP1360A and Ky226, designated as population A and B, respectively (Horn et al. 2015). For this purpose, RILs heterozygous for the QTL interval but homozygous for the rest of the genome were selected. RILs were selfed and their offspring, the HIF populations A and B, were genotyped, using 39 SNP-based Kompetitive allele-specific polymerase chain reaction (KASP™) markers (see below). From the HIF populations, genotypes that were homozygous recombinant in the QTL for phenotyping (see below) were selected. This selection resulted in the derivation of 83 homozygous genotypes from P092 x FAP1360A (population A) and 102 homozygous recombinants from Ky226 x P092 (population B). These individuals were selfed to generate seeds for replicated infection experiments as described below. Heterozygous HIF offspring were subjected to another round of selfing.

### DNA extraction, KASP™ marker design, and application

DNA was extracted using an in-house protocol. About 25 to 50 mg of frozen plant material was homogenized using Tissue Lyzer II (Qiagen, Hilden, Germany), and 150 µl extraction buffer was added. After centrifugation (10 min, 4 °C, 4.000 RCF), 75 µl supernatant was transferred to a new plate containing 60 µl isopropanol, gently mixed, and centrifuged (10 min, 4 °C, 4.000 RCF). The supernatant was discarded, and the pellet was washed with 150 µl ethanol (70%) and eluted in 100 µl TE buffer. The DNA concentration of random samples was checked with a nanophotometer.

KASP™ markers were designed in several rounds, based on different sources of SNP information. No matter the source, SNP information was filtered for identical alleles of inbreds Ky226 and FAP1360A but a different allele of P092. In the first round, molecular marker information from Horn et al. (2015) was employed. Sequences flanking the SNPs at least 50 bp upstream and downstream were retrieved from the maize genetics and genomics database (https://www.maizegdb.org/) using reference version 4 of the B73 genome. Later, information from the targeted sequencing of parental inbred lines was used for marker design. Such SNPs were preferred, which had identical sequences of P092, Ky226, and FAP1360A in the 50 bp flanking regions. We aimed to spread markers evenly across the QTL confidence interval. Sequences were sent to the manufacturer, LGC Genomics Ltd. (Hoddesdon, Herts, UK), to design the markers.

Genotyping was as recommended by the manufacturer. An ABI Quantstudio 5 (Applied Biosystems) was used for analysis.

### Inoculation and quantification of virus titer

The above-described HIF populations were evaluated for IR and EX in four replications per genotype, in which an experimental unit comprised eight to ten plants of one genotype. In all experiments, founder inbred lines FAP1360A, Ky226, and P092 but also two additional inbreds resistant and susceptible to BYDV-PAV, D408 and W64A, respectively, were used as controls. When the maize plants reached the two-leaf stage, BYDV-PAV-carrying *R. padi* were collected from the barley plants used for rearing. The aphids were then distributed manually in small groups across the maize plants so that approximately ten aphids per plant were applied. This time point was subsequently designated as the start of inoculation. After one week, plants were treated with the insecticide “Careo” (Substral Celaflor) or “Confidor” (Bayer CropScience). Six weeks after the start of the inoculation, leaf material from the sixth leaf of each plant was harvested separately, and the virus titer was determined using the DAS-ELISA method as described by Horn et al. (2013).

The IR was calculated as the percentage of plants of one experimental unit with virus titer ≥ 0.5. EX was calculated as the mean virus titer per experimental unit.

### Phenotypic data analyses and genetic mapping

Estimated marginal means of EX and IR across all replications of an experiment were calculated using the following mixed linear model:

$$Y_{ij} = \, \mu \, + \, g_{i} + \, r_{j} + \, e_{ij}$$

where Y_ij_ was the phenotypic observation for the ith genotype for the jth replicate, μ the general mean, g_i_ the effect of the ith genotype, r_j_ the effect of the jth replication, and e_ij_ the residual.

With the same model but with genotype as a random effect, genotypic σ_g_^2^ and error variance σ_e_^2^ were calculated. Broad-sense heritability on an entry mean basis (H^2^) was calculated.

To associate the above described marginal means of each genotype of the HIF populations with the molecular marker profile, the following linear model was used for each of the 39 SNP-based KASP™ markers:

$$Y_{ik} = \, \mu \, + \, s_{k} + \, m_{i} + \, e_{ik}$$

where s_k_ was the effect of the HIF population and m_i_ that of the marker, which significance was tested with an F-test. In a second mapping approach, we used the phenotypic data for BYDV-PAV resistance of an association panel from Horn et al. (2014). HapMap3.2.1 genotypic data (Bokowski et al. 2018) corresponding to the 300 Kbp QTL confidence interval were retrieved from the PANZEA website. Ambiguous data points were removed and sequence variants were filtered for minor allele frequency > 0.025 and missing values < 20%. Association analysis was conducted as described by Horn et al. (2014) using the Q matrix from Flint-Garcia et al. (2005) and the K matrix from Horn et al. (2014).

The analyses were conducted using R version 3.6.3 (R Core Team 2020, https://www.R-project.org/) with packages “lme4” version 1.1–23 (Bates et al 2015), “emmeans” version 1.5.1 (Lenth 2020), “car” version 3.0–10 (Fox and Weisberg 2019), and RStudio version 1.3.1073 (RStudio Team 2020, http://www.rstudio.com/).

Protein sequences and information on gene annotation were retrieved from the maize genetics and genomics database (https://www.maizegdb.org/). Protein sequences were loaded into InterPro (https://www.ebi.ac.uk/interpro/; Paysan-Lafosse et al. 2022) to predict functional protein domains.

#### Degree of dominance of the resistance gene

Six sub-populations of HIFs were created to estimate the degree of dominance. Each of them consisted of one genotype homozygous for the allele of P092 at marker SYN4811 and a sibling homozygous for the allele of Ky226 or FAP1360A. Additionally, one sibling was included, which was heterozygous at marker SYN4811. Alternatively, a heterozygous genotype was created by crossing two homozygous siblings.

Plants were inoculated with BYDV-PAV carrying *R. padi* in two replications. The virus titer was measured, and EX and IR per genotype were calculated (see previous section). The mean EX and IR per group (homozygous resistant, homozygous susceptible or heterozygous) were calculated and the degree of dominance estimated.

#### Genomic characterization of founder maize inbreds

### Probe design

Probes for target enrichment sequencing of founder inbred lines were designed for the QTL confidence interval identified by Horn et al. (2014) plus 1 Mbp to the distal end of the chromosome. At the time of probe design, reference sequences of seven maize inbred lines were available: B73 (Zm00001d.2), CML247 (Zm00024a.1), EP1 (Zm00010a.1), F7 (Zm00011a.1), Mo17 (Zm00014a.1), PH207 (Zm00008a.1), and W22 (Zm00004a.1). The probe design team of the manufacturer (Roche/Nimblegen) used these sequences. Repetitive sequences were masked, and 2 million probes were designed in which up to three matches to the reference genome of B73 were allowed.

### DNA extraction and sequencing

DNA was extracted using the NucleoMag Plant Kit (Macherey & Nagel GmbH & Co. KG Düren, Germany), following the manufacturer’s instructions. The DNA concentration and quality were assessed with a nanophotometer, a Qubit fluorometer (Invitrogen) with a Qubit dsDNA HS Assay Kit, and a Fragment Analyzer (Advanced Analytical Technologies).

Sample preparation was conducted following PacBio protocol “Multiplex Genomic DNA Target Capture Using SeqCap EZ Libraries” (PN 100–893-500 version 03). In brief, genomic DNA was fragmented using gTUBES (Covaris), end-repaired, and A-tailed using a KAPA HyperPlus Kit (Roche Sequencing Solutions). It was then barcoded, and adapters were ligated. DNA fragments were then amplified, using a universal primer (Sigma-Aldrich) and Takara LA Taq DNA polymerase hot-start version (Takara). PCR fragments were size-selected for fragment length greater than 4.5 kbp with a BluePippin™ automated DNA size selection device (Sage Science), pooled, hybridized with SeqCap EZ Prime Developer Probes (Roche Diagnostics) and captured using HyperCap Target Enrichment Kit (Roche Diagnostics), and Dynabeads M-270 Streptavidin (Invitrogen by Thermo Fisher Scientific Baltics). The captured DNA fragments were amplified, using a universal primer and Takara LA Taq DNA polymerase hot-start version (Takara). The SMRTbell™ library preparation was performed per the manufacturer’s instructions. The sequencing was conducted on a Sequel II platform (PacBio) to deliver highly accurate long reads appropriate for the identification of structural variants.

### Data processing, SNP calling, and structural variant (SV) prediction

B73 reference sequence version 5 became available after wet lab analyses were finished. We decided to use version 5 as a reference for the data processing workflow despite using version 4 during the target enrichment, as the former was the most recent version of the sequence and, thus, is expected to have a higher quality.

Obtained reads were demultiplexed with Python package demultiplex and trimmed with bbmap (sourceforge.net/projects/bbmap/). Trimmed reads were used for a reference-guided assembly of the QTL confidence interval with RaGOO (Alonge et al. 2019). They were mapped to B73 reference sequence version 5 (Zm00001eb) using minimap2 (Li et al. 2018) with parameter -ax asm20 and the coverage was calculated with SAMtools as well as custom AWK and Python scripts. From the reads that mapped to the QTL interval, SNPs and insertions/deletions of less than 50 bp length (InDels) were called using FreeBayes (Garrison et al. 2012). SNPs and InDels were subjected to variant-effect prediction, using the Variant-Effect Predictor tool from Gramene (https://ensembl.gramene.org/Oryza_sativa/Tools/VEP#) that employs the scale-invariant feature transform (SIFT) algorithm (Ng and Henikoff 2003).

Insertions and deletions ≥ 50 bp were defined as structural variations (SVs) and were called by re-mapping reads to the B73 genome with restrictive parameters and exploiting cuteSV (Jiang et al. 2020).

#### Genome-wide gene expression analysis

### RNA extraction and sequencing

Two independent experiments were conducted to assess gene expression differences between the maize founder inbreds upon infection with BYDV. In both experiments, plants of inbreds FAP1360A, P092, and W64A were treated with BYDV-PAV-carrying *R. padi*, virus-free *R. padi*, or without aphids as control. Approximately ten BYDV-PAV-carrying aphids per plant were applied when plants reached the two-leaf stage. After one week, all the plants, including controls, were sprayed with the insecticide “Careo” (Substral Celaflor).

In experiment 1, samples were obtained 24 and 96 h post-infection (hpi). An experimental unit consisted of 4–8 plants of an inbred x treatment x timepoint of sampling combination. All plants of an experimental unit were pooled, and the experiment was replicated four times. In experiment 2, 2–4 single plants per inbred x treatment combination were tested individually. Samples were taken two weeks after inoculation.

Leaves were harvested, frozen immediately in liquid nitrogen, and stored at − 80 °C until further analysis. RNA was extracted with TRIzol (Ambion by Life Technologies) and Direct-zol RNA MiniPrep Kit (Zymo Research; experiment 1) or RNeasy Plant Mini Kit (Qiagen) following the manufacturer’s recommendations. All samples were treated with RNase-free DnaseI (ThermoFisher Scientific).

RNA concentration was quantified, using a Qubit fluorometer (Invitrogen) and a Qubit RNA HS Assay kit (Life Technologies), and the quality was assessed with a nanophotometer.

The RNA was paired-end sequenced with 150 bp reads on an Illumina (experiment 1) or DNBseq™ (experiment 2) platform, respectively.

The BYDV infection status was confirmed via DAS-ELISA six weeks after inoculation from the sixth leaf (experiment 1) or two weeks after inoculation from the youngest fully developed leaf (experiment 2).

### RNAseq data processing

RNAseq reads were filtered, including removing adaptor sequences, contamination and low-quality reads from raw reads. The unpaired reads were discarded. Exon and splice site information was retrieved from B73 reference genome version 5. Reads were aligned to this reference genome, using the HISAT2 version 2.1.0 (Kim et al. 2019). SamTools version1.6 (Li et al. 2009; Danecek et al. 2021) was used to index, sort, and filter mapped reads. Duplicates were removed. The reads per gene were counted with HTSeq version 0.11.1 (Anders et al. 2015; Putri et al. 2022).

### Analysis of differently expressed genes

Differently expressed genes (DEGs) were identified with edgeR version 3.28.1 (Robinson et al. 2010), where the following contrasts were considered: Aphid-infested plants versus Control (Aphid_vs_Ctrl), BYDV-infected plants versus Control (BYDV_vs_Ctrl), and BYDV-infected plants versus aphid-infested plants (BYDV_vs_Aphid).

The lists of DEGs were subjected to Gene Ontology (GO) and Kyoto Encyclopedia of Genes and Genomes (KEGG) enrichment analysis using ShinyGO 0.76.3 (Ge et al. 2020). Pathway databases “KEGG”, “GO Biological Process”, “GO Cellular Component”, and “GO Molecular Function” were used and parameters were set to FDR = 0.05, Pathway size: min = 2 and max = 2000, and redundancy was removed. No background gene list was provided because ShinyGO 0.76.3 employs protein-coding genes as default.

## Results

### Mapping of the BYDV-PAV resistance gene

For fine mapping of the BYDV-PAV resistance in maize, homozygous genotypes were selected, which were recombinant in the QTL confidence interval. This selection procedure resulted in 83 genotypes originating from selfings of two RILs derived from P092 x FAP1360A (population A) and 102 individuals from selfings of RILs derived from Ky226 x P092 (population B). These 185 homozygous recombinants were subjected to phenotyping for BYDV-PAV resistance.

Broad-sense heritability (*H*^*2*^) was estimated as 0.89 for EX and 0.82 for IR across the homozygous recombinants of both populations. When both populations were analyzed separately, H^2^ of population A was slightly lower, with 0.79 for EX and 0.70 for IR, compared to 0.92 for EX and 0.85 for IR in population B.

**Fig. 1:**
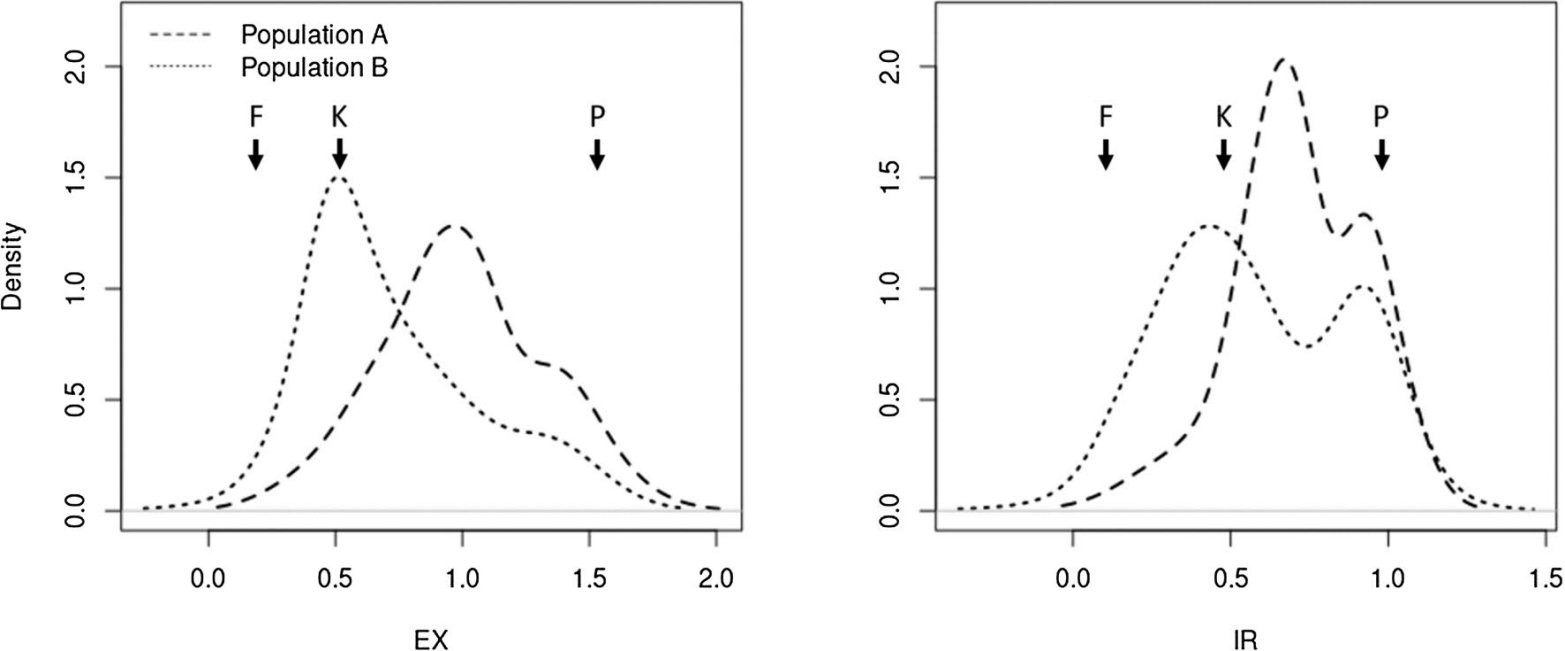
Density curves of estimated marginal means of virus titer (EX, left) and infection rate (IR, right), separated by population. One-hundred and eighty-five homozygous offspring from two heterozygous inbred families and the parental lines FAP1360A (F), Ky226 (K), and P092 (P) were infected with BYDV-PAV, and virus titer was analyzed six weeks after infection

Estimated marginal means ranged from 0.11 to 1.65 for the trait EX and -0.05 to 1.14 for the trait IR. For both traits, estimated marginal means followed a continuous distribution (Fig. 1). For a subset of genotypes, for which heterozygote siblings or progenies were available, the degree of dominance was estimated to be -0.44 for EX and -0.18 for IR, across all sub-populations and replications.

**Fig. 2:**
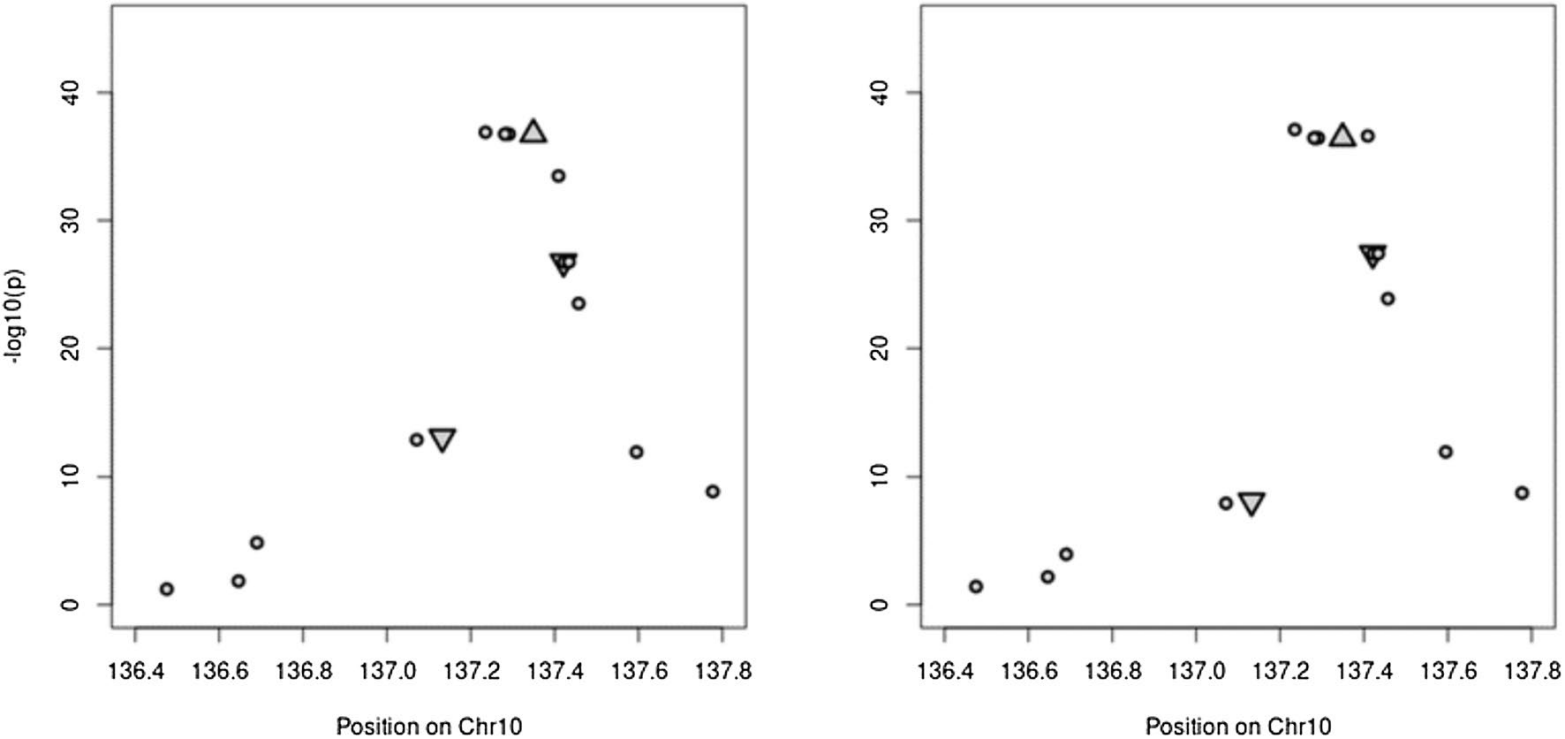
Manhattan plots for the association between BYDV-PAV resistance in 185 maize inbreds and genotypic marker. Left: trait virus titer (EX), left: trait infection rate (IR). Marker positions are provided, based on the reference sequence of B73, version 5. Up-facing triangle: marker SYN4811. Down-facing triangles: flanking marker PZE-110080306 (left) and BYDV-M20 (right)

The statistical test associating the genotyping profiles of the 185 homozygous recombinants with their marginal means for EX and IR resulted in the fine mapping of the resistance factor to the genome region between marker PZE-110080306 and the newly developed BYDV-M20 as a flanking marker of the QTL interval, as for them, the slope of the trendline changed the direction (Fig. 2). Furthermore, the markers were significantly associated also after correcting for multiple testing. The physical position of these markers delimits the resistance factor to the region between 137,131,915 and 137,409,058 bp on chromosome 10, which comprises nine genes. These genes are Zm00001eb428020 (GRMZM2G018027), a candidate gene for BYDV-PAV resistance identified by Horn et al. (2014), two transcription factors (Zm00001eb427970 and Zm00001eb427980), a putative WAK-related receptor-like protein kinase family protein (Zm00001eb427960), a putative RING zinc finger domain superfamily protein (Zm00001eb427950), a P-loop containing nucleoside triphosphate hydrolases superfamily protein (Zm00001eb428010), and three genes of unknown function (Zm00001eb427940, Zm00001eb427990, and Zm00001eb428000) (Table 1). Separate analyses of populations A and B revealed that the resistance genes from FAP1360A and Ky226 are located in the same genomic region corresponding to the previously mentioned interval.

**Table 1:** Genes in the ~ 0.3 Mbp long QTL confidence interval for BYDV-PAV resistance in maize on chromosome 10. Start and end positions are based on the reference sequence of B73, version 5

Gene ID	Start	End	Description / suggested function
Zm00001eb427940	137,133,463	137,134,445	unknown
Zm00001eb427950	137,197,560	137,198,870	RING zinc finger domain superfamily protein
Zm00001eb427960	137,214,761	137,217,464	WAK-related receptor-like protein kinase family protein
Zm00001eb427970	137,229,874	137,233,357	ABI3-VP1-transcription factor 2
Zm00001eb427980	137,263,651	137,266,456	Transcription factor bHLH28 like
Zm00001eb427990	137,278,582	137,280,136	unknown
Zm00001eb428000	137,280,991	137,284,148	unknown
Zm00001eb428010	137,285,187	137,290,824	DNA2/NAM7 helicase-like protein
Zm00001eb428020	137,348,959	137,349,907	response to oxidative stress, response to cadmium ion

**Fig. 3:**
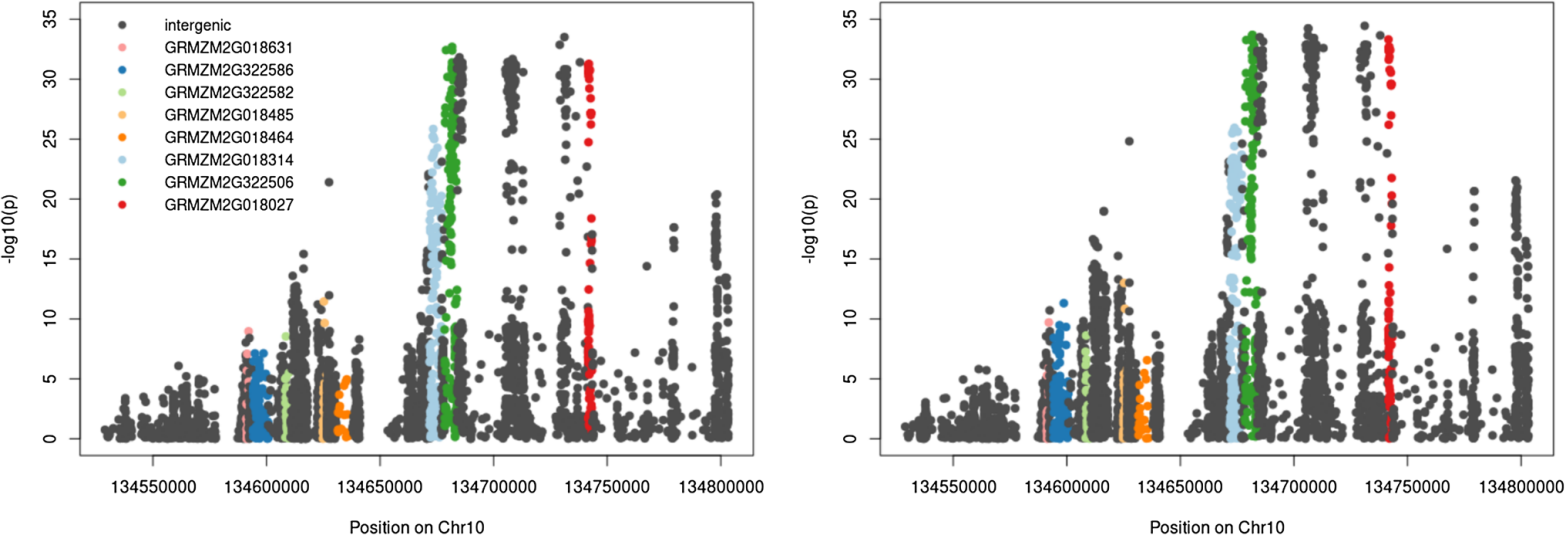
Manhattan plots for the association analysis of BYDV-PAV resistance and HapMap3.2.1 marker. The association of genetic markers with IR (left) and EX (right) is shown. Variant positions and gene names are given based on B73 reference genome version 3

To further reduce the number of candidate genes, we used an association mapping approach based on the BYDV-PAV phenotyping data from an association mapping panel described by Horn et al. (2014) and the genotypic data from HapMap3.2.1 (Bokowski et al. 2018). The strongest association for BYDV-PAV resistance was found for sequence variants located in genes GRMZM2G322506 (Zm00001eb428010) and GRMZM2G018027 (Zm00001eb428020) and the intergenic space in between these two (Fig. 3).

### Analysis of sequence variation in the QTL interval

The targeted long-read sequencing of the five founder inbred lines FAP1360A, Ky226, P092, D408, and W64A resulted in 1,579,826 raw reads and 8,421,284,727 bases sequenced. Reads were filtered and mapped against the B73 reference genome version 5 (Zm00001eb) and assembled to contigs, whose total length was between 9,747,441 and 14,948,168 bp per inbred.

The three BYDV-PAV-resistant inbreds had similar numbers of variants compared to reference B73. We counted 1972, 1911, and 1869 SNPs and InDels for D408, FAP1360A, and Ky226, respectively. The BYDV-PAV-susceptible inbred W64A had slightly fewer variants (1797), and for BYDV-PAV-tolerant inbred P092, the lowest number of variants (1139) compared to B73 was detected.

SNPs and InDels were subjected to variant-effect prediction. More than 94% of SNPs and InDels were predicted to be modifiers, such as upstream and downstream-gene variants, intron variants, intergenic variants, and 5`- and 3`-UTR variants (Table 2). The SIFT algorithm predicted a high impact for 19 SNPs and InDels. However, of those, only one SNP, which leads to a frameshift in gene Zm00001eb428000, was shared by the three BYDV-PAV-resistant founder inbreds but not by P092 and W64A (Supplementary Table 1). Additionally, six protein-altering variants were detected. One protein-altering variant in gene Zm00001eb428010 was shared by the three BYDV-PAV-resistant founder inbreds but not by P092 and W64A. The other five variants were located in gene Zm00001eb427970, of which three were shared between D408 and FAP1360A, and two were unique to Ky226.

**Table 2:** Variant-effect prediction of single nucleotide polymorphisms (SNPs) and InDels (< 50 bp) in the ~ 0.3 Mbp QTL confidence interval on chromosome 10 between 137,131,915 and 137,409,058 bp (B73 ref v5) of five founder inbred lines

Impact	Consequence	Count
High	stop gained	2
	start lost	1
	stop gained, frameshift variant	1
	frameshift variant	12
	splice-acceptor variant, coding-sequence variant	1
	splice-acceptor variant, intron variant	2
Moderate	protein-altering variant	6
	inframe deletion	6
	inframe insertion	4
	missense variant, splice-region variant	1
	missense variant	159
Low	splice-region variant, intron variant	11
	splice-region variant, synonymous variant	1
	stop-retained variant	1
	synonymous variant	85
Modifier	5 prime UTR variant	63
	3 prime UTR variant	148
	intron variant	244
	upstream-gene variant	1688
	downstream-gene variant	1258
	intergenic variant	1699

Additionally, 34 SVs were detected in the 0.3 Mbp QTL confidence interval, the majority of them (24) unique to one inbred (Supplementary Table 2). Only nine SVs were located in a gene. Remarkably, the three BYDV-PAV-resistant inbreds shared a 54 bp deletion in the 5`-UTR of gene Zm00001eb428010, a 91 bp insertion in intron 6, and a 362 bp deletion in intron 7 of the same gene. These were not present in susceptible and tolerant genotypes, respectively.

Only SNPs and InDels with low (synonymous variants) or modifier effect (intron or upstream/downstream gene variants) but no SNPs with predicted high or protein-altering effect or SVs were detected for the BYDV-PAV-resistance candidate gene Zm00001eb428020.

### Gene expression

Two independent experiments were conducted to analyze the effect of BYDV-PAV infection on genome-wide gene expression in maize. Samples were taken at 24 hpi and 96 hpi in experiment 1 as well as two weeks after inoculation in experiment 2. Only a small number of genes was significantly differently expressed in experiment 1 (Table 3) among treatments. P092 had the most DEGs with 111 DEGs for all time points and comparisons. For FAP1360A, 88 DEGs were found, but none for W64A. However, we did not find any DEGs in FAP1360A for the comparison BYDV_vs_Aphid. Most DEGs were found among treatments in the upregulated group at 96 hpi in both FAP1360A and P092. Remarkably, the 25 downregulated genes in BYDV_vs_Ctrl of FAP1360A at 24 hpi were enriched for nucleotide and nucleoside biosynthesis and metabolism processes.

**Table 3:** Counts of differently expressed genes (DEGs) in experiment 1 at 24 and 96 h past infection (hpi) and 2 weeks past infection (wpi) in experiment 2

Genotype	Comparison	24 hpi		96 hpi		2 wpi	
		up	down	up	down	up	down
FAP1360A	BYDV vs Control	4	25	26	0	347	290
	BYDV vs Aphid	0	0	0	0	0	0
	Aphid vs Control	0	1	31	1	18	36
P092	BYDV vs Control	0	1	61	5	2904	3509
	BYDV vs Aphid	0	1	43	0	3058	3546
	Aphid vs Control	0	0	0	0	350	383
W64A	BYDV vs Control	0	0	0	0	4577	4880
	BYDV vs Aphid	0	0	0	0	5137	5010
	Aphid vs Control	0	0	0	0	3671	4001

In the second experiment, a considerably higher number of DEGs was detected (Table 3). *R. padi* infestation and BYDV-PAV infection had a low effect on gene expression in FAP1360A in comparison to P092 and W64A. We found eight to 19 times more DEGs in P092 and 13 to 204 times more DEGs in W64A than in FAP1360A, respectively. Interestingly, BYDV_vs_Aphid in FAP1360A had no DEGs. In contrast, BYDV_vs_Aphid was the comparison with most DEGs in P092 and W64A for upregulated and downregulated genes, respectively. Among the downregulated genes in P092 in BYDV_vs_Aphid, KEGG pathways “Phagosome” (zma04145) and “Spliceosome” (zma03040) were enriched 2.8-fold and 1.9-fold.

Only two genes of the 0.3 Mbp QTL confidence interval—Zm00001eb428010 and Zm00001eb428020—were expressed in both experiments. Additionally, Zm00001eb428000 was expressed in experiment 2 but with a lower abundance than Zm00001eb428010 and Zm00001eb428020. None of these three genes was differently expressed in any genotype in any treatment combination.

## Discussion

BYD is one of the economically most important diseases in small-grain cereals (Choudhury et al. 2017; van den Eynde et al. 2020). Increasing autumn and winter temperatures are expected to aggravate the BYD problem (Pidon et al. 2024). For maize, BYD infection has a direct negative effect on different phenotypic characters (Beuve et al. 1999; Loi et al. 2004; Panayotou 1977). Additionally, maize plays an important role in the BYD transmission cycle, serving as a “green bridge” between the harvest of small-grain cereals in early summer and the sowing of winter cereals in autumn (Brown et al. 1984; Haack et al. 1999; Rashidi et al. 2021). The cultivation of BYD-resistant maize is expected to reduce BYD pressure on maize and small-grain cereals, such as wheat and barley. Different viruses cause BYD, of which BYDV-PAV is the most prevalent virus worldwide. The breeding of BYDV-PAV-resistant maize is strongly facilitated by the availability of markers closely linked to the resistance gene. Furthermore, the cloning of this BYDV-PAV resistance QTL will be also informative for the breeding of BYD-resistant barley and wheat genotypes by providing targets for mutagenesis experiments. Therefore, the BYDV-PAV QTL identified by Horn et al. (2015) was fine-mapped in our study.

### Fine mapping of the BYDV-PAV resistance in maize

To avoid the potential problem related to marker-trait associations due to population structure (Stich et al. 2008), our study exploited HIF populations. Despite the observed high heritabilities around 0.8, the marginal means of the homozygous recombinants in the HIF populations showed no distinct categories for the virus-titer phenotypes EX and IR but a continuous distribution trending toward a bimodal distribution (Fig. 1). The reasons for this observation are the heritabilities lower than one together with a limited difference in the virus-titer phenotypes EX and IR between resistant and susceptible/tolerant genotypes. Therefore, an ANOVA approach was used in our study to fine-map the resistance factor. Furthermore, as we observed a difference in virus-titer phenotypes EX and IR between both HIF populations (Fig. 1), we fitted a population effect in our linkage analyses of BYDV-PAV titer.

These analyses allowed to reduce the QTL confidence interval from 8 Mbp (Horn et al. 2015) to ~ 0.3 Mbp (Fig. 2). The interval comprised nine annotated genes in the fifth version of the B73 reference genome (Zm00001eb) (Table 1). The putative functions of these nine genes suggest that some of them might be involved in virus defense-related processes and, thus, convey resistance against BYDV-PAV in maize. However, as these links were rather weak, we performed an association study using BYDV-PAV resistance data from an association mapping population (Horn et al. 2014) and HapMap3.2.1 genotypic data (Bokowski et al. 2018) for the 0.3 Mbp QTL confidence interval to further reduce the number of candidate genes. When Horn et al. (2014) performed the association analysis, the density of SNP markers in the BYDV-PAV resistance QTL was relatively low. Thus, no markers were located in the genes surrounding GRMZM2G018027. In contrast, the HapMap3.2.1 genotypic data used in our study shows a high density and multiple markers are located in every gene of the 0.3 Mbp QTL confidence interval (Fig. 3). Our analysis showed strong associations of BYDV-PAV resistance with sequence variants located in genes Zm00001eb428010 and Zm00001eb428020, but not with sequence variants located in other genes of the 0.3 Mbp QTL confidence interval. This finding confirms that either Zm00001eb428010 or Zm00001eb428020 confers BYDV-PAV resistance in maize.

### Two candidate genes in the QTL for BYDV-PAV resistance in maize

The protein encoded by Zm00001eb428010 contains two AAA domains. GO-terms for this gene are RNA binding (GO:0003723) and helicase activity (GO:0004386). AAA domain containing proteins possess diverse functions, including the disassembly of SNARE proteins, protein quality control, DNA replication, ribosome assembly, and viral replication (Khan et al. 2022). The protein encoded by Zm00001eb428010 is predicted to belong to the DNA2/NAM7-like helicase family. Nam7, also known as Upstream frameshift 1 (Upf1), targets plant and animal viruses for nonsense-mediated mRNA decay (for review, see May and Simon 2021). However, many viruses escape Upf1-mediated decay through *cis*-acting RNA sequences and *trans*-acting viral proteins (May and Simon 2021).

Horn et al. (2014) identified three SNPs in Zm00001eb428020 (GRMZM2G018027), which were significantly associated with EX and IR and proposed it as a candidate gene for BYDV-PAV resistance in maize. Zm00001eb428020 is associated with GO-terms”response to oxidative stress “ (GO:0006979) and”response to cadmium ion “ (GO:0046686) in the molecular function category and”nuclear speck “ (GO:0016607) in the cellular component category.

Nuclear speckles are nuclear membraneless bodies enriched in splicing factors (Hasenson and Shav‐Tal 2020). Fungal effectors can induce the susceptibility of host plants by inducing alternative splicing of host transcripts at nuclear speckles (Tang et al. 2022). The same process is suspected for oomycete effectors (Wang et al. 2015).

The best BLAST hit for Zm00001eb428020 in *Arabidopsis thaliana* is the gene *OXS3* (Horn et al. 2014). *OXS3* is expressed during response reactions to oxidative stress (Blanvillain et al. 2009) and likely improves resistance to the Tobacco mosaic virus in *A. thaliana* by producing hydrogen peroxide (Wang and Culver 2012).

In both RNAseq experiments, Zm00001eb428010 and Zm00001eb428020 were the only two genes in the 0.3 Mbp QTL confidence interval, which were expressed, indicating that either one of them is the causative agent for BYDV-PAV resistance in maize. However, neither Zm00001eb428010 nor Zm00001eb428020 was differently expressed among the different treatments, suggesting that BYDV-PAV resistance in maize might act at time points that were not covered by our experiments. The more likely explanation is that the difference between resistant and susceptible/tolerant genotypes appears at the protein level and not at the gene expression level. Protein abundance might be shaped by post-transcriptional gene regulation (for review, see Prall et al. 2019). Protein substrate specificity and kinetics might be influenced by changes in amino acid sequence evoked through SNPs or alternative splicing. Indeed, alternative splicing has been shown for maize upon viral infection (Du et al. 2020; Zhou et al. 2022).

Additionally, three SV were observed for the candidate gene Zm00001eb428010. In contrast to most other detected SV in the QTL confidence interval were these three SVs shared between all three BYDV-PAV resistant inbreds but not present in susceptible and tolerant inbreds when the sequences were compared to reference B73. B73 is a BYDV-PAV susceptible inbred. Thus, sequence variations between B73 and the resistant genotypes absent in the tolerant and susceptible genotypes are potentially associated with resistance. The relatively small size of the SVs in Zm00001eb428010 (54 bp, 91 bp, and 362 bp) is in accordance with findings by Hufford et al. (2021). Two SVs were located in the intronic regions of Zm00001eb428010 and one 54 bp deletion was located in the 5`-UTR (untranslated region). Some 5`-UTRs are known to influence translation efficiency (Yamasaki et al. 2018). Generally, 5`- and 3`-UTRs possess *cis*-acting elements for post-transcriptional control, which regulate mRNA stability, transport, and translation efficiency, as well as the functioning and subcellular localization of the translated proteins (Mignone et al. 2002). Thus, the deletion in the 5`-UTR may influence protein abundance and/or properties. Thus, we speculate that variants in Zm00001eb428010 may influence the encoded protein. However, further work on the protein-altering effect is necessary to identify isoforms of Zm00001eb428010 expressed in different inbreds or under different conditions and to analyze differences in protein substrate specificity and kinetics.

Zm00001eb428010 and Zm00001eb428020 are located at the distal end of the maize chromosome 10, a genomic region that contains multiple overlapping QTL for resistance to diverse viruses (Redinbaugh et al. 2018). This suggests that the BYDV-PAV resistance gene may be efficient against other maize viruses as well.

### Changes in genome-wide gene expression following BYDV-PAV infection

In contrast to other gene expression studies on BYD infection in cereals or virus infection in maize (Cao et al. 2019; Li et al. 2018; Rong et al. 2018; Shen et al. 2020; Wang et al. 2013; Zhou et al. 2016), only a low number of DEGs was detected (Table 3). We suspect that early reactions to BYDV-PAV infection are limited to the phloem cells penetrated by aphids during feeding and maybe a few adjacent cells. Using whole leaves might have led to “dilution effects” that prevent the detection of DEGs because unaffected cells outnumber infected cells. Thus, single-cell sequencing might be a more feasible approach.

Experiment 2 represents processes in the plant at a later infection stage in systemic leaves compared to experiment 1. The virus titer corresponded to the number of DEGs. Very low virus titer and numbers of DEGs were detected in the BYDV-PAV-resistant inbred FAP1360A when compared to BYDV-PAV-tolerant P092 and BYDV-PAV-susceptible W64A. Together with the fact that no DEGs were found in BYDV_vs_Aphid (Table 3), this leads to the conclusion that the BYDV-PAV resistance gene may act at early stages after infection, hampering virus replication and/or movement, enabling the plant to grow relatively unaffected.

A lower number of genes of BYDV-PAV-tolerant inbred P092 were differently expressed compared to the BYDV-PAV-susceptible inbred W64A (Table 3), which potentially reflects the lack of symptom formation (Horn et al. 2013; this study). Consistently, DEGs of the BYDV-PAV tolerant genotype P092 were not enriched for genes related to chloroplasts or photosynthesis. This might be a starting point to answer the question of why BYDV-PAV can replicate and spread in P092 but does not cause visible symptoms.

## Conclusion

Combining biparental mapping, association mapping, gene expression profiling, and targeted sequencing, we identified two candidate genes for BYDV-PAV resistance in maize: Zm00001eb428010 and Zm00001eb428020. The predicted functions of these genes suggest a rather nonspecific resistance mechanism, potentially by interfering with virus replication or induction of ROS signaling. BYDV-PAV infection did not influence the expression of Zm00001eb428010 and Zm00001eb428020 in any inbred. However, sequence variants of Zm00001eb428010 present in BYDV-PAV-resistant inbreds but absent in BYDV-PAV-susceptible or BYDV-PAV-tolerant inbreds suggest that abundance and/or properties of the proteins encoded by Zm00001eb428010 may lead to BYDV-PAV resistance. Providing closely linked markers to this gene strongly facilitates the selection of resistant material. Finally, orthologs of these two genes in barley, wheat, and other cereals are promising targets for mutagenesis experiments to generate BYDV-resistant genotypes.

### Supplementary Information

Below is the link to the electronic supplementary material.

Supplementary file1 (DOCX 20 kb)

## Data Availability

The original sequencing datasets will be uploaded upon the acceptance of the manuscript.
