# Supplementary material for: Fine mapping a QTL for BYDV-PAV resistance in maize
Source: Theor Appl Genet. 2024 Jun 19;137(7):163. doi: 10.1007/s00122-024-04668-z (PMC11186928; doi:10.1007/s00122-024-04668-z)
Supplement: Supplementary file 1 — Supplementary file1 (DOCX 20 kb) [file 122_2024_4668_MOESM1_ESM.docx]

Supplementary Table 1: Single Nucleotide Polymorphisms (SNPs) and InDels (< 50 bp) with high impact and selected moderate impact consequences in the ~0.3 Mbp long QTL confidence interval on chromosome 10 of five maize inbreds.

| Location | Gene | Genotype | Impact | Consequence |
| --- | --- | --- | --- | --- |
| 137132252-137132263 | Zm00001eb427930 | FAP1360A | high | frameshift variant |
| 137132285-137132296 | Zm00001eb427930 | FAP1360A | high | frameshift variant |
| 137132357-137132362 | Zm00001eb427930 | FAP1360A | high | frameshift variant |
| 137134227-137134229 | Zm00001eb427940 | FAP1360A, W64A | high | stop gained |
| 137134415-137134415 | Zm00001eb427940 | W64A | high | stop gained |
| 137198057-137198068 | Zm00001eb427950 | D408, W64A | high | splice-acceptor variant, coding-sequence variant |
| 137215832-137215843 | Zm00001eb427960 | W64A | high | splice-acceptor variant, intron variant |
| 137216497-137216501 | Zm00001eb427960 | W64A | high | frameshift variant |
| 137231759-137231768 | Zm00001eb427970 | D408, FAP1360A | moderate | protein-altering variant |
| 137231849-137231853 | Zm00001eb427970 | Ky226_DNA | moderate | protein-altering variant |
| 137232026-137232029 | Zm00001eb427970 | D408, FAP1360A | moderate | protein-altering variant |
| 137232639-137232669 | Zm00001eb427970 | Ky226_DNA | moderate | protein-altering variant |
| 137232639-137232669 | Zm00001eb427970 | Ky226 | high | frameshift variant |
| 137232649-137232669 | Zm00001eb427970 | W64A | high | frameshift variant |
| 137232655-137232667 | Zm00001eb427970 | FAP1360A | high | frameshift variant |
| 137232747-137232755 | Zm00001eb427970 | D408, FAP1360A | moderate | protein-altering variant |
| 137265234-137265242 | Zm00001eb427980 | W64A | high | frameshift variant |
| 137280016-137280026 | Zm00001eb427990 | P092, W64A | high | frameshift variant |
| 137281005-137281005 | Zm00001eb428000 | W64A | high | start lost |
| 137281019-137281020 | Zm00001eb428000 | D408, FAP1360A, Ky226 | high | frameshift variant |
| 137283154-137283160 | Zm00001eb428000 | Ky226 | high | frameshift variant |
| 137283470-137283474 | Zm00001eb428000 | W64A | high | splice acceptor variant, intron variant |
| 137285376-137285385 | Zm00001eb428010 | FAP1360A | high | stop gained, frameshift variant |
| 137287706-137287718 | Zm00001eb428010 | D408, FAP1360A, Ky226 | moderate | protein-altering variant |
| 137288545-137288548 | Zm00001eb428010 | D408, FAP1360A, Ky226 | moderate | missense variant, splice-region variant |
| 137290395-137290403 | Zm00001eb428010 | FAP1360A | high | frameshift variant |

Supplementary Table 2: Structural variants (SVs) in five maize inbreds in the ~0.3 Mbp long QTL confidence interval on chromosome 10 compared to the B73 reference sequence. DEL: deletion, INS: insertion, DUP: duplication. The size is in bp.

|  |  |  | D408 | |  | FAP1360A | |  | Ky226 | |  | P092 | |  | W64A | |
| --- | --- | --- | --- | --- | --- | --- | --- | --- | --- | --- | --- | --- | --- | --- | --- | --- |
| Position | Gene | Type | length | reads |  | length | reads |  | length | reads |  | length | reads |  | length | reads |
| 137134930 | intergenic | DEL | -69 | 12 |  | -69 | 10 |  | - | - |  | - | - |  | - | - |
| 137196587 | intergenic | DEL | -68 | 99 |  | -69 | 50 |  | - | - |  | -68 | 67 |  | - | - |
| 137217634 | intergenic | DEL | - | - |  | - | - |  | - | - |  | -71 | 13 |  | - | - |
| 137222506 | intergenic | INS | 525 | 695 |  | 522 | 434 |  | - | - |  | - | - |  | - | - |
| 137223407 | intergenic | INS | - | - |  | - | - |  | 367 | 36 |  | - | - |  | - | - |
| 137231044 | Zm00001eb427970 exon 2 | DUP | - | - |  | - | - |  | 586 | 33 |  | - | - |  | - | - |
| 137233263 | Zm00001eb427970 exon 2 | DEL | - | - |  | - | - |  | -35 | 52 |  | - | - |  | - | - |
| 137233396 | Zm00001eb427970 promoter | DEL | - | - |  | - | - |  | - | - |  | -45 | 31 |  | - | - |
| 137233519 | intergenic | INS | 298 | 70 |  | 299 | 32 |  | - | - |  | - | - |  | - | - |
| 137234264 | intergenic | INS | - | - |  | - | - |  | 713 | 10 |  | - | - |  | - | - |
| 137234364 | intergenic | INS | - | - |  | - | - |  | - | - |  | 126 | 31 |  | - | - |
| 137235051 | intergenic | INS | - | - |  | - | - |  | - | - |  | - | - |  | 30 | 7 |
| 137236873 | intergenic | INS | 332 | 22 |  | - | - |  | - | - |  | - | - |  | - | - |
| 137246382 | intergenic | INS | - | - |  | - | - |  | 48 | 49 |  | - | - |  | - | - |
| 137246779 | intergenic | DEL | - | - |  | - | - |  | -44 | 40 |  | - | - |  | - | - |
| 137269727 | intergenic | DEL | -998 | 86 |  | -998 | 51 |  | - | - |  | - | - |  | - | - |
| 137276225 | intergenic | DUP | - | - |  | - | - |  | 917 | 26 |  | - | - |  | - | - |
| 137277113 | intergenic | INS | - | - |  | - | - |  | 189 | 27 |  | - | - |  | - | - |
| 137277265 | intergenic | DEL | - | - |  | - | - |  | -84 | 22 |  | - | - |  | - | - |
| 137278264 | intergenic | INS | - | - |  | - | - |  | - | - |  | - | - |  | 366 | 235 |
| 137279395 | Zm00001eb427990 intron 1 | DEL | - | - |  | - | - |  | - | - |  | -35 | 207 |  | - | - |
| 137279469 | Zm00001eb427990 intron 1 | INS | - | - |  | - | - |  | - | - |  | - | - |  | 187 | 488 |
| 137283830 | Zm00001eb428000 intron 5 | INS | 1623 | 20 |  | - | - |  | - | - |  | - | - |  | - | - |
| 137284303 | intergenic | INS | - | - |  | - | - |  | - | - |  | - | - |  | 260 | 235 |
| 137284911 | intergenic | DEL | - | - |  | - | - |  | - | - |  | - | - |  | -50 | 290 |
| 137285231 | Zm00001eb428010 5` UTR | DEL | -54 | 552 |  | -54 | 239 |  | -54 | 175 |  | - | - |  | - | - |
| 137289267 | Zm00001eb428010 intron 6 | INS | 91 | 77 |  | 91 | 44 |  | 91 | 25 |  | - | - |  | - | - |
| 137289894 | Zm00001eb428010 intron 7 | DEL | -362 | 84 |  | -362 | 50 |  | -361 | 29 |  | - | - |  | - | - |
| 137293457 | intergenic | DEL | - | - |  | -18838 | 109 |  | -18838 | 66 |  | - | - |  | -18840 | 131 |
| 137293458 | intergenic | DEL | -18838 | 206 |  | - | - |  | - | - |  | - | - |  | - | - |
| 137316117 | intergenic | DEL | -187 | 32 |  | -187 | 23 |  | -187 | 14 |  | - | - |  | - | - |
| 137316902 | intergenic | DEL | -2766 | 202 |  | -2767 | 117 |  | -2766 | 67 |  | - | - |  | - | - |
| 137320044 | intergenic | DEL | -17947 | 172 |  | -17947 | 95 |  | -17947 | 61 |  | - | - |  | - | - |
| 137385285 | intergenic | DEL | -17869 | 65 |  | -17869 | 41 |  | -17869 | 25 |  | - | - |  | - | - |
